# CCL18-induced LINC00319 promotes proliferation and metastasis in oral squamous cell carcinoma via the miR-199a-5p/FZD4 axis

**DOI:** 10.1038/s41419-020-02978-w

**Published:** 2020-09-18

**Authors:** Xiao Jiang, Jingpeng Liu, Simin Li, Bo Jia, Zhijie Huang, Jun Shen, Haiyun Luo, Jianjiang Zhao

**Affiliations:** Stomatological Hospital, Southern Medical University, Guangzhou, Guangdong China

**Keywords:** Oral cancer, Cell biology

## Abstract

Long non-coding RNAs (lncRNAs), which may be modulated by chemokines, are key regulators in many cancers including oral squamous cell carcinoma (OSCC). An understanding of lncRNAs involved in chemokine (CC motif) ligand 18 (CCL18)-induced OSCC promotion remains elusive. The present study using lncRNA sequencing found LINC00319 to be significantly upregulated in OSCC cells subjected to rCCL18 stimulation. Furthermore, LINC00319 knockdown was found to attenuate the carcinogenic function of CCL18 in OSCC, reducing OSCC proliferation, metastasis, epithelial-mesenchymal transition (EMT), and angiogenesis. LINC00319 was demonstrated to act as a ceRNA in OSCC, which directly responded to miR-199a-5p and rescued the repression of FZD4 by miR-199a-5p. Functionally, in vitro and in vivo experiments showed that LINC00319 promoted OSCC growth and metastasis via downregulating miR-199a-5p and upregulating FZD4. In vitro rescue assays demonstrated that miR-199a-5p inhibitor or FZD4 overexpression reversed the effects of LINC00319 silencing in OSCC. Importantly, the expression of miR-199a-5p and FZD4 were found to be mediated by CCL18, and miR-199a-5p mimics inhibited the CCL18-promoting effects in oral cancer cells. Taken together, these results evidenced a mechanism of CCL18 action in OSCC mediated through the LINC00319/miR-199a-5p/FZD4 signaling pathway, which may comprise a potential target for OSCC therapeutic development.

## Introduction

Oral squamous cell carcinoma (OSCC) is the most common malignant tumor occurring in the oral and maxillofacial region. Despite improvements in diagnosis and therapy, the 5-year survival rate of OSCC patients remains <50%^1^. Rapid proliferation, high vascularization, and distant metastasis are the primary characteristics of OSCC progression. Numerous studies have revealed that OSCC development is a multi-factorial and multi-step process which involves the expression of both coding and non-coding genes^2,3^. However, detailed molecular mechanisms underpinning the biological behavior of OSCC remain unclear.

Chemokine (C–C motif) ligand 18 (CCL18), a member of the CC subgroup of chemokines, is involved in the tumor microenvironment and plays a critical role in several cancers including OSCC^4–7^. Our previous study revealed that CCL18 was overexpressed in OSCC and could enhance the growth and metastasis of oral cancer by activating the PI3K/AKT signaling pathway^8^. In addition, the exogenous CCL18 stimulation was reported to promote the metastasis and induce the acquisition of stem cell characteristics of OSCC cells by activating the mTOR-slug pathway^9^. Apart from regulating these pathways mentioned above, increasing evidence also showed that the chemokines involved in cancer development were targeted by several long non-coding RNAs (lncRNAs). For example, CCL21 induces the overexpression of lncRNA MALAT1, thereby further activating the mTOR signaling pathway and promoting cutaneous lymphoma cell migration^10^. In addition, LINC00092 was shown to act as a downstream molecular effector of CXCL14 in ovarian cancer^11^. The CXCL12/CXCR4 axis was found to promote the inflammatory infiltration of colon cancer by regulating the lncRNA XIST/miR-133a-4p/RhoA signaling axis^12^. In particular, CCL18 was shown to mediate the lncRNA HOTAIR/miR-130a-5p/ZEB1 axis in esophageal squamous cell carcinoma^13^. However, lncRNAs putatively involved in CCL18-driven OSCC malignancy have not been investigated.

In general, LncRNAs, which are longer than 200 nucleotides in length, have no protein-coding capacity, and was associated with inflammation, tumorigenesis, and metastasis. Many dysregulated lncRNAs (e.g., LncRNA-p23154 and LncRNA HCP5) are understood to affect OSCC progression^14,15^. Among all the OSCC-dysregulated lncRNAs, the specific lncRNA which can target the CCL18 in OSCC needs to be selected and used for further investigation. The primary research at the earliest of this study performed the lncRNA sequencing assay and found that CCL18 stimulation in OSCC caused the dysregulation of LINC00319, confirming LINC00319 to be the downstream target of CCL18. Recent data has revealed that dysregulated LINC00319 was implicated in the progression of many cancers by involving a ceRNA network. In nasopharyngeal carcinoma, LINC00319 overexpression expedites cancer cells metastasis by sponging miR-1207-5P, resulting in the de-repression of the oncogene KLF12^16^. In addition, the LINC00319/miR-4492/ROMO1 axis was found to facilitate the proliferation and invasiveness of bladder cancer^17^. However, whether LINC00319 is implicated in OSCC by involving a specific ceRNA network remains to be unexplored.

The present study hypothesized that the knockdown of LINC00319 could inhibit the oncogenic function of CCL18 in OSCC, and therefore, aimed to investigate CCL18-induced LINC00319 expression as a potential molecular mechanism underpinning OSCC.

## Materials and methods

### Cell culture and OSCC tissues

The HSC6 cell line was obtained from CinoAsia co., Ltd (shanghai, China). CAL27, SCC9, and human oral keratinocytes (HOK) cell lines were purchased from TongPai biotechnology co., LTD (shanghai, China). 293T embryonic kidney cell line and Human Umbilical Vein Endothelial Cell line (HUVEC) were bought form ATCC (Manassas, VA, USA). All cells were identified by STR profiling and were negative for mycoplasma. OSCC cells, HEK293T cells, and HUVEC were maintained in Dulbecco’s modified Eagle’s medium (DMEM, Gibco, Grand Island, NY, USA) supplemented with 10% fetal bovine serum (FBS, Gibco, USA) and 1% penicillin-streptomycin. HOK cells were cultured in KSFM (Gibco, USA). Cells were incubated in a humidified atmosphere of 5% CO_2_ at 37 °C. Recombinant human CCL18 (rCCL18) was purchased from Peprotech (Peprotech, Inc.,Princeton, NJ, USA).

Primary OSCC tissues (*n* = 10) and some adjacent normal tissues (*n* = 10) were obtained postoperatively. All patients provided written informed consent before study entry. The study was approved by the Ethics Committee of Stomatological Hospital, Southern Medical University.

### lncRNA sequencing

Total RNA of rCCL18-stimulated HSC6 cells was extracted using TRIzol reagent (Invitrogen, USA). A total RNA-seq library was prepared using the VAHTS Total RNA-seq (H/M/R) Library Prep Kit (Vazyme, China) and Illumina HiSeq^TM^2000 (Illumina, USA) sequencing was performed to capture the expression of lncRNA and mRNA. The subsequent data were processed using HISAT comparison software. lncRNAs and mRNAs with *P* < 0.05 were identified as differentially expressed.

### Cell transfection and stable cell lines

LINC00319 siRNA sequences, miR-199a-5p mimics and miR-199a-5p inhibitor and their corresponding controls were designed by GenePharma (Jiangsu, China) and transfected into HSC6 and CAL27 cells with Lipofectamine 2000 (Invitrogen, USA). Transfection efficiency was verified by qRT-PCR after 48 h of transfection. Lentivirus treated with LNC00319 interference and FZD4 overexpression were designed (Genechem, Shanghai, China), and were used to infected HSC6 and CAL27 cells for 72 h. In all, 2.5 μg/ml puromycin (Thermo Fisher Scientific, Waltham, MA, USA) was utilized for screening stable cells. The sequences of LINC00319 RNAi, miR-199a-5p mimics, and miR-199a-5p inhibitor are presented in supplementary Table 1.

### Dual-luciferase reporter assays

Wild-type (WT) LINC00319, Mutant-type (MUT) LINC00319, WT-3′-UTR-FZD4 and MUT-3′-UTR-FZD4 were cloned into PmirGLO vector (GenePharma, China) to generate luciferase receptor plasmids (GenePharma, China). HEK-293T cells were cultured into 24-well plates with the cell density at 70%. Luciferase receptor plasmids, blank vector plasmid, miR-199a-5p mimics, and miR-199a-5p NC were co-transfected into HEK-293T cells using lipofectamine 2000 (Invitrogen, USA) regent. The luciferase activity was detected by a dual-luciferase reporter assay system (Promega Corp., Madison, WI, USA).

### Fluorescence in situ hybridization assays

Fluorescence in situ hybridization assay was used to ascertain the cellular localization of miR-199a-5p and LINC00319. An RNA FISH probe mix for LINC00319 with Cy3-labeling and a FISH probe mix for miR-199a-5p with FAM-labeled were each designed (GenePharma, China), and applied to HSC6 cells using a RiboTM Fluorescent In Situ Hybridization Kit (RiboBio, Guangzhou, China) according to kit instructions. HSC6 cells were plated in 24-well culture plates at a concentration of 6 × 10^4^ per well. Cells were fixed with 4% paraformaldehyde, permeabilized with 0.3% Triton X-100, and blocked with a pre-hybridization buffer. In all, 4 μmol/L FISH probes for miR-199a-5p and LINC00319 were added on HSC6 cells with the hybridization buffer and incubated at 37 °C overnight. Thereafter, cell nuclei were cunterstained with 4,6-diamidino-2-phenylindole (DAPI) and the images were obtained with laser scanning confocal microscopy (Carl Zeiss AG, Germany).

### Western blot analysis

Cells were lysed using cell lysis buffer containing phosphatase inhibitor, protease inhibitor, and PMSF (KeyGEN BioTECH, Jiangsu, China). In all, 20 μg protein per lane were separated by 10% SDS-PAGE, transferred to PVDF membrane (Merck KGaA, Darmstadt, Germany), and incubated with the following primary antibodies at 4 °C overnight: E-cadherin, N-cadherin, ZEB2, GAPDH, VEGF-A, MMP-9, and FZD4. Next, the PVDF membrane was incubated with secondary antibody at room temperature, followed by ECL reagent (Telenbiotech, Guangzhou, China) for chemiluminescent detection. The source and dilution of antibodies were listed in Supplementary Table 2.

### Immunofluorescence

HSC6 and CAL27 cells were digested to prepare a suspension and plated. When cells reached 70–80% confluence, adherent cells were fixed with 4% paraformaldehyde solution for 10 min, permeabilized with 0.3% Triton X-100 for 5 min, and blocked with 5% bovine serum albumin (BSA) for 1 h. Subsequently, the cells were incubated with primary antibodies: E-cadherin and N-cadherin overnight at 4 °C. After rinsing with PBS, these were then incubated with secondary antibody in the dark for 1 h and then counterstained with DAPI (Invitrogen, Carsbad, CA, USA) for 5 min. Images were then obtained using an automated upright microscope system (Leica, DM4000B Leica Microsystems, Wetzlar, Germany). The source and dilution of antibodies were listed in Supplementary Table 2.

### Immunohistochemistry

The xenografts tumor tissues of nude mice, were dewaxed in xylene and rehydrated in a graded alcohol series. The morphological of tissues was observed by using HE staining. Immunohistochemistry (IHC) was performed using the antibodies of E-cadherin, N-cadherin, ZEB2, VEGF-A, MMP-9, and FZD4, and visualized using a CFDA immunohistochemical test kit (Gene Tech, Shanghai, China). In addition, the expression of FZD4 in the OSCC tissues and adjacent normal tissues was detected by IHC. Photographed were obstained using an automated upright microscope system (Leica, DM4000B Leica Microsystems, Wetzlar, Germany). The source and dilution of primary antibodies were listed in Supplementary Table 2.

### qRT-PCR analysis

Cells were collected, and total RNA was extracted with TRIzol reagent (Invitrogen, USA). Reverse transcription of miRNA was performed using miRcute miRNA First-strand cDNA kit (TIANGEN Biotech, Beijing, China). miRcute miRNA qPCR Detection Kit (SYBR Green; TIANGEN Biotech, China) was used to determine the mRNA expression levels of miRNA. The thermocycling conditions were as follows: 2 min at 94 °C, 40 cycles of 20 s at 94 °C, and 34 s at 60 °C. U6 was used as an endogenous controls.

For the qRT-PCR detection of mRNA, complementary DNA (cDNA) was synthesized using FastKing gDNA Dispelling RT SuperMix kit (TIANGEN, China). qPCR was performed using a Talent qPCR PreMix kit (TIANGEN, China). The thermocycling conditions were as follows: 3 min at 95 °C, followed by 40 cycles of 5 s at 95 °C and 15 s at 60 °C. The relative levels of mRNA expression were determined by normalizing to GAPDH expression levels using the 2^-∆∆Cq^ method. The primers sequences used are listed in Supplemantary Table 3.

### CCK8 assay

In total, 5000 cells were seeded in 96-well plate for 24 h, 48 h, and 72 h. The CCK8 regent (Sigma-Aldrich, Louis, MO, USA) was added as per the kit protocol, and the absorbance values at 450 nm for each well were determined using a microplate reader (Thermo Fisher Scientific. Waltham, MA, USA).

### Flow cytometry assay

OSCC cells transfected with siLINC00319 and siNC were cultured with 20 ng/ml rCCL18, the flow cytometry assay was used to detected cell cycle and cell apoptosis. For cell cycle assay, cells were collected and fixed with ethanol overnight at 4 °C. After washed with PBS, the cells were treated with 500 μl PI/RNase A (KeyGen, Jiangsu, China) at 37 °C for 30 min, and analyzed by flow cytometry. For apoptosis assay, cells were collected and washed with PBS twice. In all, 500 μl Annexin V binding buffer was used to resuspend the cells. Then 5 μl Annexin V-APC and 5 μl PI (KeyGen, China) were added into the mix buffer and incubated for 10 min at room temperature. The samples were analyzed by flow cytometry immediately.

### Transwell assay

Cell migration and invasion were determined using 8 µm pore filters in a transwell assay (Corning, New York, NY, USA), performed according to the manufacturer’s instructions. The upper chamber was precoated with 50 μl 20% Matrigel (Gibco, USA) for invasion assay. After appropriate treatment, the cells were seeded in the upper insert, and the lower chamber was filed with DMEM containing 15% FBS. After 24 h of incubation at at 37 °C with 5% CO_2_, the cells that migrated to the lower chambers were fixed by 4% formaldehyde and stained with 0.4% crystal violet. Five fields of view at ×50 magnification were randomly selected and photographed using a light microscope (Carl Zeiss AG, Oberkochenm Germany).

### Angiogenesis assay

OSCC cells were pretreated according to different experimental groups. HUVEC cells were cultures for 48 h. Thereafter, the supernatants of OSCC cells and HUVEC cells were removed and replaced with a serum-free medium for 24 h. These conditioned media were collected and mixed in equal proportion. In all, 8 × 10^4^ HUVEC cells were seeded into matrigel (BD, CA, USA) precoated 48-well plates and incubated with mixed media for 4 h. The cultures were then photographed using a light microscope (Carl Zeiss AG, Oberkochenm Germany). The number of vessel branches, rings, nodes and area of rings were analyzed using Image J software (Rasband, NIH, USA).

### In vivo transplanted tumors in nude mice

Immunodeficient female BALB/c nude mice (4–6 weeks old) were purchased form Animal Care Unit of Guangdong (Guangdong, China), and were randomly divided into two groups (5 mice/group). To establish the xenografts model, HSC6 cells (5 × 10^7^, 100 μl) stably transfected with shLINC00319 or shNC were injected into the right armpit of nude mice. The body weight of mice and tumor volume (mm^3^) was measured every seven days. Afterward, the nude mice were euthanized, and the tumor tissues were surgically removed for further analysis. The animal experiment was approved by the Institutional Animal Experiment Committee of Southern Medical University.

### Statistical analysis

The data obtained were summarized as means ± SD based on three replicates per group and analyzed using GraphPad Prism 7.00 software (GraphPad Software, Inc., La Jolla, CA, USA). A two-tailed unpaired Student’s *t* test was used for two-group samples comparisons, and one-way ANOVA were used for multi-group sample comparisons. *P* < 0.05 was considered statistically significant.

## Results

### CCL18 mediates the proliferation, metastasis, and angiogenesis of OSCC by LINC00319

**Fig. 1:**
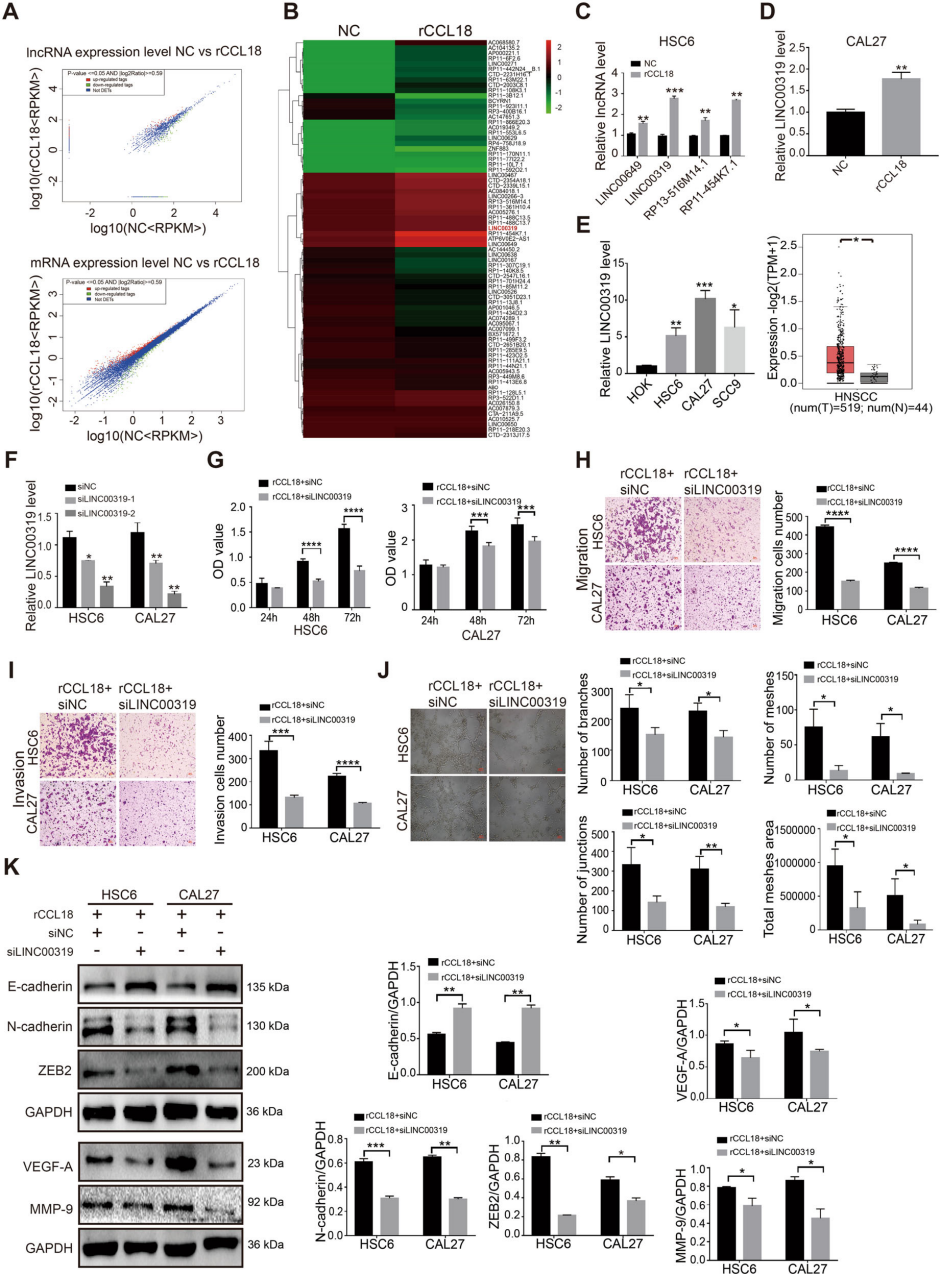
CCL18 promotes OSCC progression by regulating LINC00319. **a** Scatter plot of differentially expressed lncRNAs and mRNAs in CCL18-stimulated HSC6 cells, which was determined by lncRNA sequencing. **b** Heatmaps of differentially expressed lncRNAs. **c** qRT-PCR detected four upregulated lncRNAs (i.e., LINC00649, LINC00319, RP13-516M14.1, and RP11-454K7.1) in rCCL18-stimulated HSC6 cells. **d** qRT-PCR detected the expression level of LINC00319 in rCCL18-stimulated CAL27 cells. **e** qRT-PCR detected the expression level of LINC00319 in OSCC cells and HOK cell (*n* = 3), and TCGA database analyzed the expression of LINC00319 in HNSCC tissues. **f** The effective siLINC00319 fragments were screened in HSC6 cells and CAL27 cells by qRT-PCR (*n* = 3). **g** CCK8 assay detected the proliferation in rCCL18-stimulated OSCC cells with or without siLINC00319 silencing (*n* = 3). **h**, **i** Transwell assay evaluated the migration (**h**) and invasion (**i**) of cells described in **g** (*n* = 5). **j** Angiogenesis assay described the angiogenesis of HUVEC cells (i.e., number of branches, meshes, junctions, and total meshes area) which co-cultured with the supernatant of OSCC cells described in **g** (*n* = 3). **k** The protein level of EMT and angiogenesis markers in cells described in **g** was detected using western blotting (*n* = 3). *P < 0.05; **P < 0.01; ***P < 0.001; ****P < 0.0001.

By using lncRNA sequencing, 191 differentially expressed lncRNAs (*P* < 0.05) and 660 differentially expressed mRNA (*P* < 0.05) were identified in rCCL18-stimulated HSC6 cells (Fig. 1a). Based on the heatmaps of these differentially expressed lncRNAs (Fig. 1b), we selected four significantly upregulated lncRNAs (RP11-454K7.1, LINC00319, LINC00649, and RP13-516M14.1) to be verified by qRT-PCR and noted that their expressions were all found to have increased under the rCCL18 stimulation, where LINC00319 was the most upregulated lncRNA (Fig. 1c). The upregulation of LINC00319 was also verified in rCCL18-stimulated CAL27 cells (Fig. 1d). The LINC00319 mRNA levels was examined in OSCC cells by performing the qRT-PCR assay. The results showed that LINC00319 mRNA levels were significantly higher in OSCC cells (HSC6, CAL27, and SCC9) than in HOK cells, which was consistent with the outcomes shown by performing the TCGA database analysis (Fig. 1e).

To explored the function of the CCL18-LINC00319 axis in OSCC, two siRNA sequences against LINC00319 were transfected into OSCC cells (HSC6 and CAL27) and screened by qRT-PCR. As shown in Fig. 1f, the siRNA-2 sequence appeared as the best-fitted siRNA for the LINC00319 knockdown. CCK8, cell cycle, and cell apoptosis were used to investigate the role of CCL18-LINC00319 axis in OSCC growth. Upon knockdown of LINC00319 expression, the proliferation-inducing effect of rCCL18 on OSCC cells (HSC6 and CAL27) was found to be reduced at 48 h and 72 h (Fig. 1g). The percentage of HSC6 cells in rCCL18 + siLINC00319 group was significantly increased in the G0/G1 phase, and the percentage of CAL27 cells in rCCL18 + siLINC00319 group showed to be notably increased in G0/G1 phase and decreased in S phase (Fig. S1a). Meanwhile, HSC6 and CAL27 cells apoptosis rate were increased in rCCL18 + siLINC00319 group compared with that in the rCCL18+siNC group (Fig. S1b).

A transwell assay was used to detect the migration and invasiveness of OSCC cells, and Fig. 1h and i depicted that the number of cells on the submembrane surface decreased despite the stimulation of rCCL18, in the presence of siLINC00319. Tumor angiogenesis is a sign of tumor growth, invasion and metastasis. HUVEC cells were cultured with cell culture supernatant of HSC6 and CAL27 cells which were pretreated with rCCL18 + siLINC00319 and rCCL18+siNC. Results showed that the angiogenesis (i.e., number of branches, meshes, junctions, and the total mesh area)of HUVEC in the rCCL18 + siLINC00319 group was markedly attenuated compared with the control (Fig. 1j). Additionally, EMT-related proteins (E-cadherin, N-cadherin, and ZEB2) and angiogenesis-related proteins (VEGF-A, MMP-9) were detected by western blot. Compared with the rCCL18+siNC group, cells in the rCCL18 + siLINC00319 group exhibited upregulated E-cadherin expression and downregulated N-cadherin, ZEB2, VEGF-A, and MMP-9 expression levels (Fig. 1k), suggesting that LINC00319 is a downstream gene target of CCL18 in OSCC.

### LINC00319 enhances OSCC proliferation, metastasis, and angiogenesis by interacting with miR-199a-5p

**Fig. 2:**
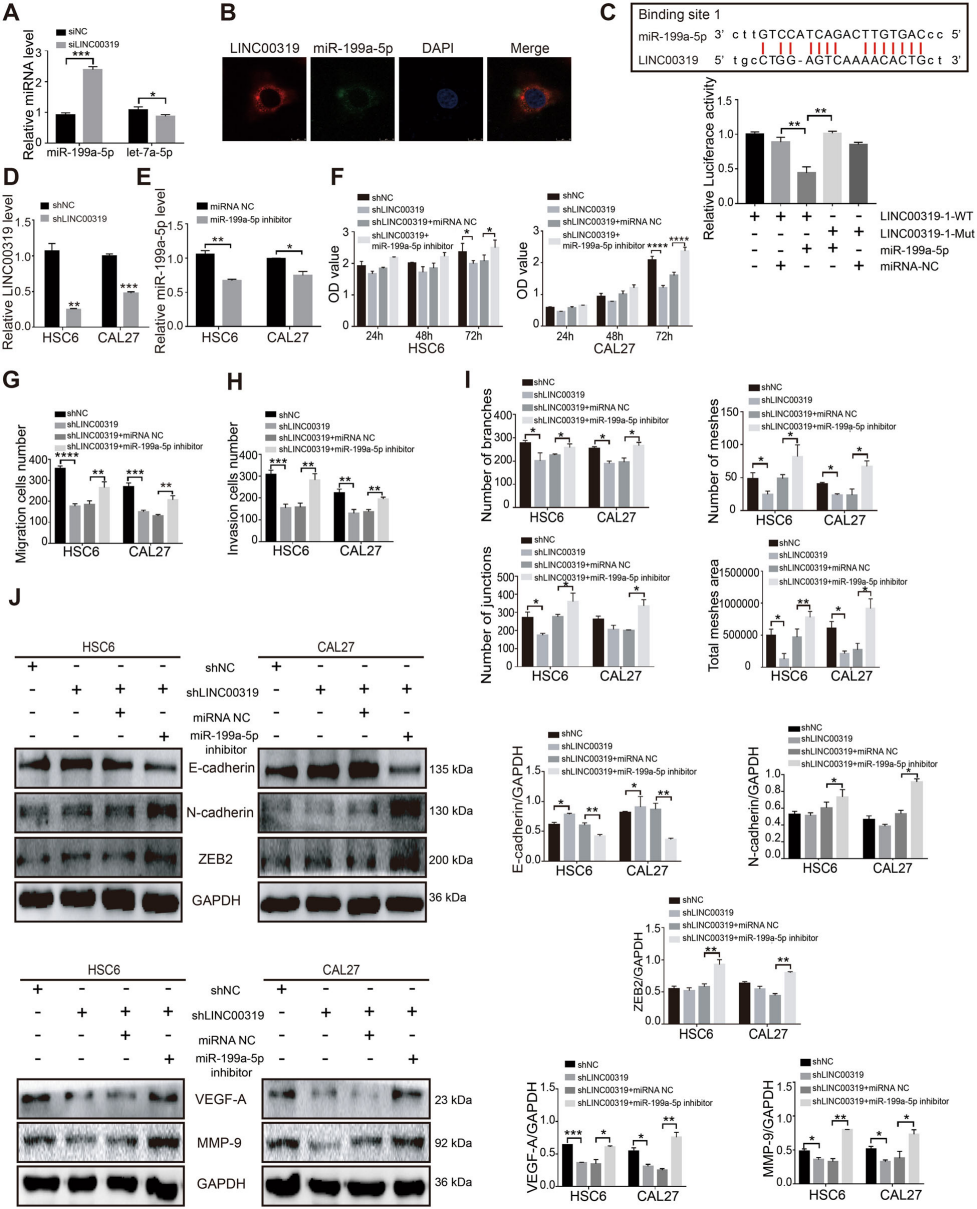
LINC00319 promotes the progression of OSCC through sponging miR-199a-5p. **a** The mRNA level of miR-199a-5p and let-7a-5p in OSCC cells with or without transfection of siLINC00319 (*n* = 3). **b** Representative FISH image of the location of LINC00319 (Red), miR-199a-5p (green), and nuclei (blue) in HSC6 cells. **c** The putative site 1 of miR-199a-5p in the LINC00319 3’UTR was analyzed by dual-luciferase reporter assays (*n* = 3). **d** The mRNA level of LINC00319 in OSCC cells infected with LINC00319-silencing lentivirus (*n* = 3). **e** The mRNA level of miR-199a-5p in OSCC cells infected with miR-199a-5p inhibitor (*n* = 3). **f** CCK8 assay detected that the proliferation of LINC00319-silenced cells with or without miR-199a-5p inhibitor, as well as the negative control group (*n* = 3). **g**, **h** Cells were treated as described in **f**. The migration cells number (**g**) and invasion cells number (**h**) in transwell assay were analyzed (*n* = 5). **i** The number of branches, meshes, junctions, and total meshes area in HUVEC cells co-cultured with the supernatant of OSCC cells described in **f** (*n* = 3). **j** The protein expression of EMT markers and angiogenesis markers in cells described in **f** was detected using western blotting (*n* = 3). *P < 0.05; **P < 0.01; ***P < 0.001; ****P < 0.0001.

LINC00319 has been reported to participate in the tumorigenesis of nasopharyngeal carcinoma, bladder cancer, and lung cancer through sponging miRNA^16–18^. We chose the miRanda database to predict miRNA that could possibly bind to LINC00319. The results indicated that miR-199a-5p and let-7a-5p may potentially bind to LINC00319 with high conservation. However, only miR-199a-5p showed a negative correlation with LINC00319 (Fig. 2a).

The FISH assay confirmed that LINC00319 and miR-199a-5p were primarily expressed in the cytoplasm, and provided a basis indicating their potential interaction (Fig. 2b). Two miR-199a-5p candidate binding sites in the 3′UTR of LINC00319 were assessed via bioinformatics analysis. Based on the putative binding site, luciferase receptor plasmids with LINC00319 wild type (WT) and LINC00319 mutant type (MUT) were constructed. And the synthetic miR-199a-5p mimic can effectively elevate the expression of miRNA in OSCC cells. Co-transfection of cells with LINC00319-WT (site 1) and miR-199a-5p mimics was noted to significantly reduce the luciferase activity as compared to transfection with miR-199a-5p NC. However, the co-transfection of cells with LINC00319-MUT (site 1) and miR-199a-5p mimics did no affect on the luciferase activity (Fig. 2c). Co-transfection of cells with both LINC00319-WT (site 2) and LINC00319-MUT (site 2) in addition to miR-199a-5p mimics also resulted in no change in luciferase activity (Fig. S2a). Taken together, these findings confirm that LINC00319 may function by absorbing miR-199a-5p.

To explore the potential effect of LINC00319 on OSCC malignancy development mediated through miR-199a-5p, we constructed OSCC cells in which LINC00319 was stably knocked down (Fig. 2d) and designed a miR-199a-5p inhibitor (Fig. 2e), which was transfected into LINC00319-silenced OSCC cells. The CCK8 assay demonstrated that OSCC cells proliferation decreased 72 h after LINC00319 silencing, and that miR-199a-5p inhibitor treatment compensated for the effect of LINC00319 knockdown on OSCC cells proliferation at 72 h (Fig. 2f). LINC00319-silencing also led to decreased migration and invasiveness of OSCC cells. When miR-199a-5p inhibitor was transfected into LINC00319-silenced OSCC cells for 48 h, the migration and invasion abilities of the cells were found improved (Fig. 2g, h and Fig. S2b,c). Subsequently, we examined the angiogenic ability of HUVEC co-culture with cancer cell supernatant from the different treatment groups described above. Compared with the shNC control group, HUVEC exhibited decreased angiogenic ability in the shLINC00319 group. When LINC00319 and miR-199a-5p were simultaneously inhibited in OSCC cells, the angiogenic ability of HUVEC was found to be enhanced in comparison with that in the shLINC00319+miRNA NC group (Fig. 2i). In addition, upon LINC00319 depletion, E-cadherin protein levels were found increased, N-cadherin and ZEB2 maintained low expression, and MMP-9 and VEGF-A protein levels were found decreased. The expression patterns of these proteins were reversed when a miR-199a-5p inhibitor was used to stimulate the shLINC00319-treated OSCC cells (Fig. 2j). Together, these in vitro experimental results confirmed the promotive function of LINC00319 binding with miR-199a-5p in oral cancer.

### MiR-199a-5p overexpression inhibits the tumorigenic function of CCL18 in OSCC

As shown in Fig. S3a, miR-199a-5p was markedly downregulated in HSC6, CAL27, and SCC9 cells compared with HOK cells. Moreover, the expression of miR-199a-5p was negatively regulated by rCCL18 in OSCC cells. (Fig. S3b). MiR-199a-5p mimic was used to evaluate the effects of miR-199a-5p on CCL18-mediated processes in OSCC (Fig. S3c). HSC6 and CAL27 cells were treated with a rCCL18+miR-199a-5p mimic and rCCL18+miRNA NC. Figure S3d showed that at 72 h, the CCL18-induced proliferation in OSCC was inhibited by miR-199a-5p upregulation. Moreover, miR-199a-5p overexpression was found to attenuate the effects of CCL18 on metastasis, EMT, and angiogenesis of OSCC (Fig. S3e, h). These results suggested that miR-199a-5p is an important component of the CCL18-mediated axis for regulating OSCC development.

### The expression of FZD4 was mediated by the LINC00319/miR-199a-5p axis

**Fig. 3:**
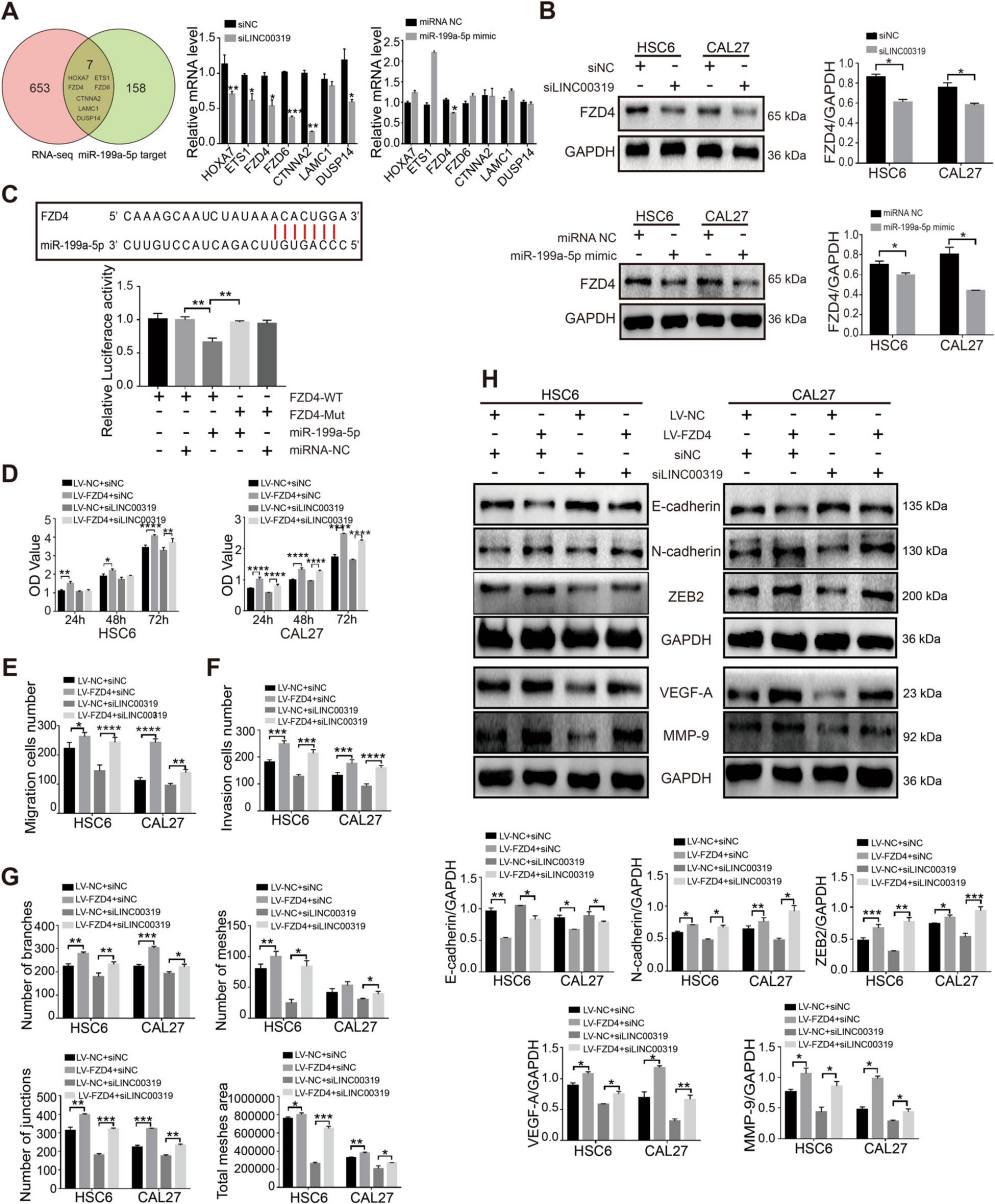
Upregulated FZD4 mediated by miR-199a-5p and LINC00319 promotes the progression of OSCC and reverses the inhibitory effects of LINC00319 silencing in OSCC cells. **a** Venn diagram identified 7 genes (i.e., HOXA7, ETS1, FZD4, FZD6, CTNNA2, LAMC1, and DUSP14) to be the overlap between lncRNA-seq results and miR-199a-5p targets. The qRT-PCR analyzed the mRNA expression of these 7 genes in HSC6 cells transfected with siLINC00319 and miR-199a-5p mimic, as well as that in corresponding control cells (*n* = 3). **b** Western blotting detected the protein expression of FZD4 in HSC6 and CAL27 cells with treated as **a** (*n* = 3). **c** The putative site of miR-199a-5p in the FZD4 3’UTR was analyzed by dual-luciferase reporter assays (*n* = 3). **d** CCK8 assay detected the proliferation of HSC6 and CAL27 cells treated in different group (LV-NC + siNC, LV-FZD4 + siNC, LV-NC + siLINC00319, and LV-FZD4 + siLINC00319; *n* = 3). **e**, **f** Cells were treated as described in **d**. The migration cells number (**e**) and invasion cells number (**f**) in transwell assay were analyzed (*n* = 5). **g** The number of branches, meshes, junctions, and total meshes area in HUVEC cells co-cultured with the supernatant of OSCC cells described in **d** (*n* = 3). **h** Western blotting evaluated the protein expression of EMT markers and angiogenesis markers in cells described in **d** (*n* = 3). *P < 0.05; **P < 0.01; ***P < 0.001; ****P < 0.0001.

To explore the downstream molecular functions of the LINC00319/miR-199a-5p axis, we screened 660 downstream mRNAs of LINC00319 from CCL18-induced lncRNA-seq (*P* < 0.05) and identified 165 downstream mRNAs of miR-199a-5p from two publicly available databases (TargetScan and David). As shown in Fig. 3a, 7 genes (HOXA7, ETS1, FZD4, FZD6, CTNNA2, LAMC1, and DUSP14) were screened out based on overlap determined using a Venn diagram. Afterward, qRT-PCR results showed that LINC00319 knockdown could effectively reduce the expression of all these genes, except LAMC1, whose difference in expression was not statistically significant. However, only FZD4 mRNA levels were decreased in the miR-199a-5p-overexpressing HSC6 cells. Western blotting further confirmed that FZD4 was positively correlated with LINC00319 and negatively correlated with miR-199a-5p (Fig. 3b). The potential binding site between FZD4 and miR-199a-5p was predicted using TargetScan. Luciferase receptor assays found that miR-199a-5p mimics suppressed the luciferase activities of the 3′-UTR of FZD4-WT, but they had no influence on the luciferase activities of the 3′-UTR of FZD4-MUT (Fig. 3c). Overall, these results implied that LINC00319, miR-199a-5p, and FZD4 constitute a ceRNA network.

Rescue assays were performed to detect the effect of FZD4 on LINC00319-mediated functions in OSCC cells. We constructed OSCC cells in which FZD4 was stably overexpressed (Fig. S4a). siLINC00319 and siNC were transfected into stable FZD4-overexpressing and NC-overexpressing OSCC cells for 48 h. The results of the CCK8 assay, transwell assay, angiogenesis assay, and western blot showed that FZD4 upregulation promoted the proliferation, migration, invasion, EMT, and angiogenesis of OSCC, and partially reverse the inhibitory those effects of siLINC00319 in OSCC (Fig. 3d–h and Fig. S4b–d).

FZD4 has been reported as an proangiogenic gene in HNSCC^19^. We also observed that the FZD4 mRNA and protein levels in OSCC cells were higher than those in HOK cells (Fig. S5a, b). IHC staining provided evidence that FZD4 was mainly expressed in the cytoplasm and cell membrane of stratum basale cells in the oral mucosa. Deeper staining for FZD4 was found in OSCC cells near the basement membrane and at the invasion front (Fig. S5c). Meanwhile, the immunofluorescence staining showed increased N-cadherin and decreased E-cadherin in FZD4 overexpression cells, which is consistent with the results of western blot assay (Fig. S5d). As LINC00319 and miR-199a-5p were both mediated by CCL18, we also detected the relationship between CCL18 and FZD4. The upregulated mRNA and protein of FZD4 were found in HSC6 and CAL27 cells stimulated with rCCL18 (Fig. S5e, f). Taken together, these results demonstrated that LINC00319/miR-199a-5p/FZD4 axis promoted the development of OSCC, and was mediated by CCL18.

### LINC00319/miR-199a-5p/FZD4 regulates OSCC malignancy *in vivo*

**Fig. 4:**
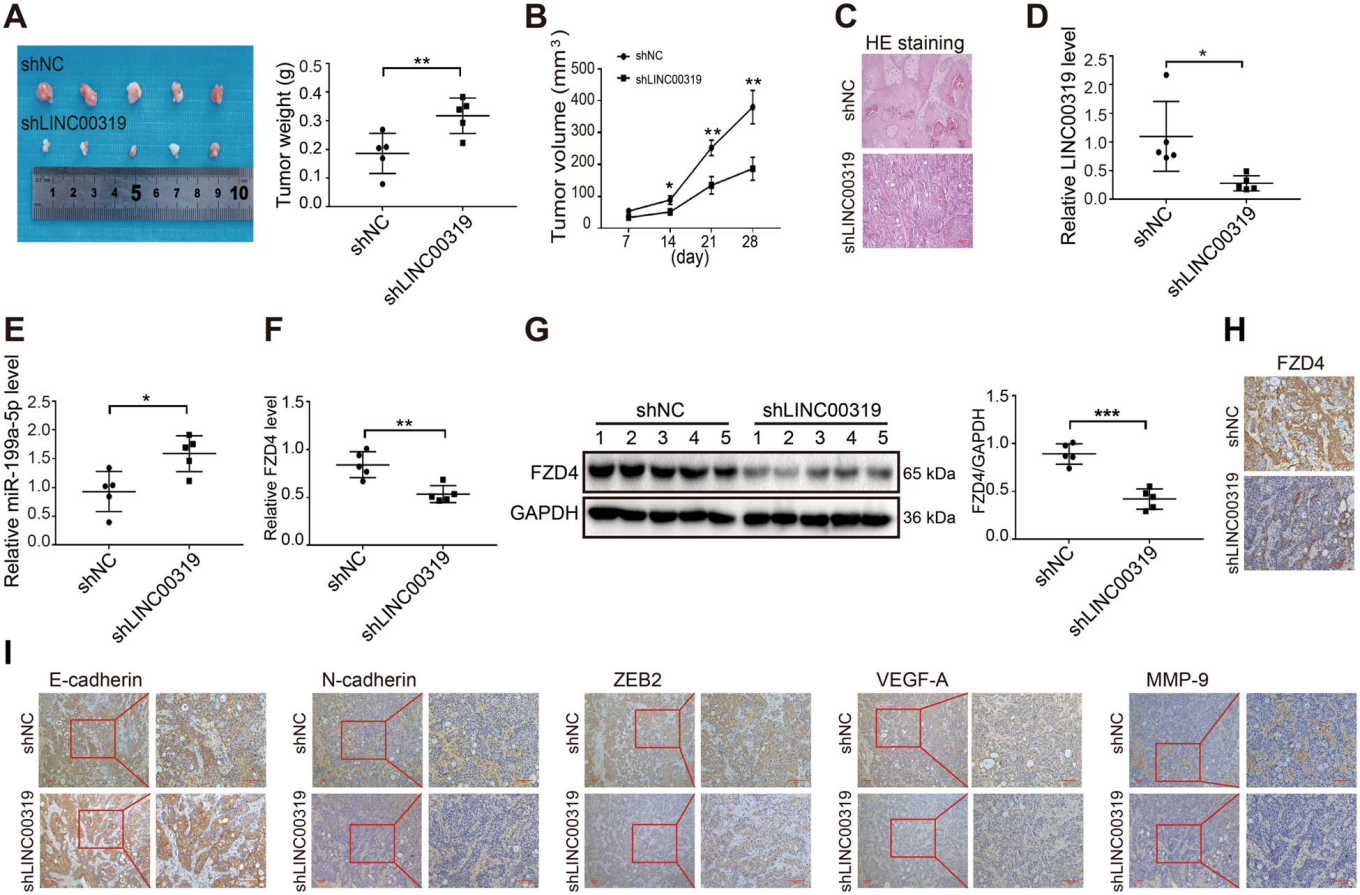
LINC00319/miR-199a-5p/FZD4 axis contributed to the malignance of OSCC in vivo. **a** The image and weight of control samples and xenograft tumor tissues formed by shLINC00319-transfected HSC6 cells (*n* = 5). **b** The volume of xenograft tumors at the time of every 7 days (*n* = 5). **c** The histomorphology of xenograft tumors with LINC00319 silencing and control was analyzed by HE staining. **d**–**f** The mRNA expression level of LINC00319 (**d**), miR-199a-5p (**e**), and FZD4 (**f**) in LINC00319-silenced xenograft tumors and control (*n* = 5). **g**, **h** The protein level of FZD4 in LINC00319-silenced xenograft tumors and control was analyzed by western blotting (**g**) and IHC (**h**) (*n* = 5). **i** The protein of EMT markers and angiogenesis markers were evaluated by IHC. *P < 0.05; **P < 0.01; ***P < 0.001.

The effect of LINC00319 on OSCC progression in vivo was evaluated using a xenograft model in BALB/c nude mice. As shown in Fig. 4a, b, tumor growth was inhibited upon LINC00319 knockdown. The shLINC00319 group exhibited a smaller tumor size and lighter tumor weight than the shNC group. HE staining showed that the cancer cells in the LINC00319-silenced group had an irregular shape, small nuclei, and displayed lesser neovasculature (Fig. 4c). qRT-PCR showed decreased expression of LINC00319 and increased expression miR-199a-5p in the LINC00319-silenced xenograft compared with that in negative control xenograft (Fig. 4d,e). Moreover, qRT-PCR, western blot, and IHC results showed the decreased expression of FZD4 on mRNA and protein level in the shLINC00319 group (Fig. 4f-h). Subsequently, EMT-associated proteins and angiogenesis-associated proteins were evaluated by IHC. Compared with the negative control group, N-cadherin, ZEB2, VEGF, and MMP-9 expression levels were found significantly reduced in the shLINC00319 group, whereas E-cadherin expression was found increased (Fig. 4i). These results further confirmed that LINC00319 promotes the growth and metastasis of OSCC, and, in sum, these results provided further evidence of an oncogenic function of the LINC00319/miR-199a-5p/FZD4 axis.

## Discussion

As a secretory protein, CCL18 is not only involved in immune and inflammatory processes, but also is known to participate in cancer development^20,21^. Our previous research reported that the abundant expression of CCL18 examined in OSCC could promote the growth and metastasis of cancer cells, and was therefore associated with the TNM stage of oral cancer patients^8^. The metastasis of cancer cells is typically accompanied by a decrease in expression levels of epithelial cell markers (e.g., E-cadherin) and an increase in expression of mesenchymal markers (e.g., N-cadherin, ZEB2, ZEB1, Vimentin, Slug and Twist, among others)^22,23^, angiogenesis-related factors (e.g., vascular endothelial growth factor (VEGF))^24^, and matrix metalloproteinases (e.g., MMP-9 and MMP-2)^25^. The exogenous CCL18 stimulation in OSCC cells was shown to cause a decrease in E-cadherin expression and an increase in N-cadherin and slug expression, suggesting that CCL18 could promote EMT in OSCC cells^26^. However, the detailed molecular mechanisms by which CCL18 may promote OSCC metastasis remain unclear.

The present study using lncRNA sequencing identified LINC00319 to be significantly upregulated in rCCL18-treated OSCC cells. LINC00319 is located in the intergenic region of chromosome 21 and acts as an oncogene in various tumors including ovarian cancer, cutaneous squamous cell carcinoma, glioma, and lung cancer^27–30^. Previously, the upregulation of LINC00319 was found to be associated with a poor prognosis of cutaneous squamous cell carcinoma patients^28^. In addition, LINC00319 was also found to accelerate tumor growth and metastasis in glioma and lung cancer^29,30^. In accordance with the expression pattern of LINC00319 shown in other types of cancers, the present study using qRT-PCR assay showed the increased level of LINC00319 mRNA in OSCC cells. In order to further verify that the function of CCL18-LINC00319 interaction pairs in OSCC progression, rCCL18 was added to cultured OSCC cells treated with siNC or siLINC00319 in vitro. When LINC00319 was silenced, the effect of CCL18 on proliferation, cell cycle, metastasis, and angiogenesis of OSCC cells was markedly inhibited, and the apoptosis inhibition of CCL18 on OSCC cells was alleviated. Moreover, compared with the rCCL18+siNC group, E-cadherin expression was increased, as well as N-cadherin, ZEB2, VEGF-A, and MMP-9 expressions were decreased in the rCCL18 + siLINC00319 group. These results evidenced that CCL18 exerts its function in OSCC through being targeted by LINC00319.

Emerging evidence indicates that LINC00319 can interfere with miRNA pathways by binding to the miRNA response element (MRE) and their interaction is involved in the regulation of many cancers. By searching the miRanda database, we found that miR-199a-5p was predicted to be a downstream target of LINC00319. In order to validate the targeting relationship between miR-199a-5p and LINC00319, luciferase reporter assays confirmed a direct interaction between LINC00319 and miR-199a-5p. The qRT-PCR results also verified the negative correlation between miR-199a-5p and LINC00319 in OSCC cells. In addition, the expression pattern and regulatory role of miR-199a-5p on OSCC development was further researched in the present study. The present study verified the dysregulation of miR-199a-5p in OSCC by showing the decreased expression of miR-199a-5p examined in HSC6, CAL27, and SCC9 cells compared with that in HOK cells. The previous researches conducted by Wei et al. showed that miR-199a-5p could suppress the migration and invasion in OSCC by targeting the EMT-related transcription factor, indicating that the tumor-suppressing role of miR-199a-5p in OSCC progression^31,32^. In accordance with the results shown in the previous study, our study also found that miR-199a-5p mimics suppressed the promoting effects of CCL18 in OSCC development; and vice versa, the miR-199a-5p inhibitor could reverse the proliferation, invasiveness, and angiogenesis of LINC00319-depleted OSCC cells. Taken together, these results demonstrated that CCL18-induced LINC00319 exerts its function in OSCC through its interaction with miR-199a-5p.

LncRNAs that interact with miRNA usually reduce the binding of miRNAs to their target genes, resulting in abnormal function of cancer cells. FZD4 was identified to be related to both LINC00319 and miR-199a-5p. Numerous studies have shown that FZD4 is a direct downstream gene of several miRNAs (e.g., miR-SNP, miR-3127-5p, miR-516b, and miR-101) and participates in key mechanisms of tumor development^33–36^. In our study, luciferase reporter assays showed that miR-199a-5p directly binds to the 3′UTR region of FZD4, and thereby inhibit the expression of FZD4. In addition, the present study also found that the overexpressed FZD4 caused by LINC00319 sponging miR-199a-5p in our study could improve the proliferation, metastasis, EMT, and angiogenesis of OSCC cells, and compensate for the suppressive effects of LINC00319 silencing in OSCC. The reason causing this phenomenon could be explained by the fact that FZD4 acts as an oncogene and can promote the tumorigenesis, development, and metastasis of various cancers^37,38^. FZD4 is associated with the activated of ERK signaling pathway and fibroblast growth factor-2 (FGF2) expression in HNSCC. Silenced FZD4 resulted in a significant decrease of p-ERK and FGF2 in HNSCC and benefited for the anti-VEGF therapy^19^. Our experiments revealed that FZD4 was overexpressed in OSCC tissues and cells. Importantly, FZD4 expression was found to be positively regulated by CCL18 in OSCC cells. Based on these findings, it is reasonable to conclude that CCL18-induced LINC00319 promotes OSCC progression through sponging miR-199a-5p and rescues the repression of FZD4 expression by miR-199a-5p.

Several limitations of this preliminary study should be acknowledged. First, a possible direct relationship between LINC00319 expression and clinical factors including the TNM stage and survival analysis were not addressed in this study owing to insufficient clinical sample size and a lack of long-term follow-up by all the included patients. It is within our further research plan to analyze OSCC patient samples and analyze the clinical significance of LINC00319 using a large cohort. In addition, FZD4 is well-known as an activator of the Wnt/β-catenin signaling pathway in several cancers^39,40^. Whether the Wnt/β-catenin signaling pathway is similarly involved, along with any other relevant pathways, in CCL18-mediated LINC00319 regulation of OSCC merits investigation in order to identify the detailed and comprehensive mechanisms operating in this context.

In conclusion, our research provides new insights into the mechanism of CCL18-mediated OSCC progression. The LINC00319/miR-199a-5p/FZD4 axis, as a ceRNA network, is an important signaling pathway through which CCL18 regulates the proliferation, invasiveness, and angiogenesis of oral cancer cells. These findings provide the preliminary basis for the LINC00319/miR-199a-5p/FZD4 axis as a novel potential therapeutic target for OSCC treatment.

## Supplementary information

Supplementary Figure legends

Supplementary Figure1

Supplementary Figure2

Supplementary Figure3

Supplementary Figure4

Supplementary Figure5

Supplementary Table 1

Supplementary Table 2

Supplementary Table 3
