# Supplementary material for: CCL18-induced LINC00319 promotes proliferation and metastasis in oral squamous cell carcinoma via the miR-199a-5p/FZD4 axis
Source: Cell Death Dis. 2020 Sep 18;11(9):777. doi: 10.1038/s41419-020-02978-w (PMC7501282; doi:10.1038/s41419-020-02978-w)
Supplement: Supplementary file 1 — Supplementary Figure legends [file 41419_2020_2978_MOESM1_ESM.docx]

**Fig.S1. CCL18 regulated cell cycle and cell apoptosis in OSCC cells.** The cell cycle (**a**) and apoptpsis (**b**) were analyzed in rCCL18-stimulated OSCC cells with or without siLINC00319 silencing (n=3). **P*<0.05; ***P*<0.01; ****P*<0.001.

**Fig.S2. LINC00319 promotes the metastasis and angiogenesis by binding miR-199a-5p. a** The putative site 2 of miR-199a-5p in the LINC00319 3’UTR was analyzed by dual-luciferase reporter assays. **b,c** The image of migration (**b**) and invasion (**c**) for LINC00319-silenced cells with or without miR-199a-5p inhibitor, as well as the negative control group (n=5). **d** The image of angiogenesis of HUVEC cells which co-cultured with the supernatant of OSCC cells described in **b,c** (n=3).

**Fig.S3. CCL18 promotes the progression of OSCC through the regulation of miR-199a-5p. a** The mRNA expression level of miR-199a-5p in OSCC cells and HOK cell (n=3). **b** The mRNA expression level of miR-199a-5p in rCCL18-sitmulated HSC6 and CAL27 cells (n=3). **c** The mRNA expession level of miR-199a-5p in HSC6 and CAL27 cells with or without miR-199a-5p mimic (n=3). **d** CCK8 assay detected the proliferation in rCCL18-stimulated OSCC cells with or without miR-199a-5p mimic (n=3). **e,f** Transwell assay evaluated the migration and invasion of cells described in **d** (n=5). **g** Angiogenesis assay examined angiogenic parameters (i.e., number of branches, meshes, junctions, and total meshes area) of HUVEC cells which were co-cultured with the supernatant of OSCC cells described in **d** (n=3). **h** The protein level of EMT and angiogenic markers in cells described in **d** was detected using western blotting (n=3). **P*<0.05; ***P*<0.01; ****P*<0.001; *****P*<0.0001.

**Fig.S4. Overexpressed FZD4 reverses the inhibitory effect of metastasis and angiogenesis in LINC00319-silenced OSCC cells. a** The protein expression level of FZD4 in HSC6 and CAL27 cells infected with FZD4 overexpression lentiviruses, negative vector as control (n=3).**P*<0.05. **b,c** The image of migration (**b**) and invasion (**c**) for HSC6 and CAL27 cells treated in the different groups (LV-NC+siNC, LV-FZD4+siNC, LV-NC+siLINC00319, LV-FZD4+siLINC00319). **d** The image of the angiogenesis of HUVEC cells co-cultured with the supernatant of OSCC cells was displayed in **b,c.**

**Fig.S5. The expression and function of FZD4 in OSCC cells. a** qRT-PCR detected the mRNA expression of FZD4 in HSC6, CAL27, SCC9 cells and HOK cell (n=3). **b** Western blotting determined the protein expression of FZD4 in HSC6, CAL27, SCC9 cells and HOK cell (n=3). **c** The FZD4 location and expression in OSCC tissue and adjacent healthy tissue were detected by IHC assay, and representative images were shown here. **d** IF assay evaluated the expression of E-cadherin (green) and N-cadherin (red) in OSCC cells with or without FZD4 overexpression. **e,f** The mRNA (**e**) and protein (**f**) expression level of FZD4 in HSC6 and CAL27 cells with rCCL18 stimulation, untreated cells as control (n=3). **P*<0.05; ***P*<0.01; ****P*<0.001; *****P*<0.0001.

**Table S1** The sequence of siLINC00319, miR-199a-5p mimic and inhibitor.

**Table S2** The antibodies used for western blot, immunofluorescence, and immunohistochemistry.

**Table S3** The primer sequences for genes used in qRT-PCR.
