# Supplementary figures and images for: CCL18-induced LINC00319 promotes proliferation and metastasis in oral squamous cell carcinoma via the miR-199a-5p/FZD4 axis
Source: Cell Death Dis. 2020 Sep 18;11(9):777. doi: 10.1038/s41419-020-02978-w (PMC7501282; doi:10.1038/s41419-020-02978-w)

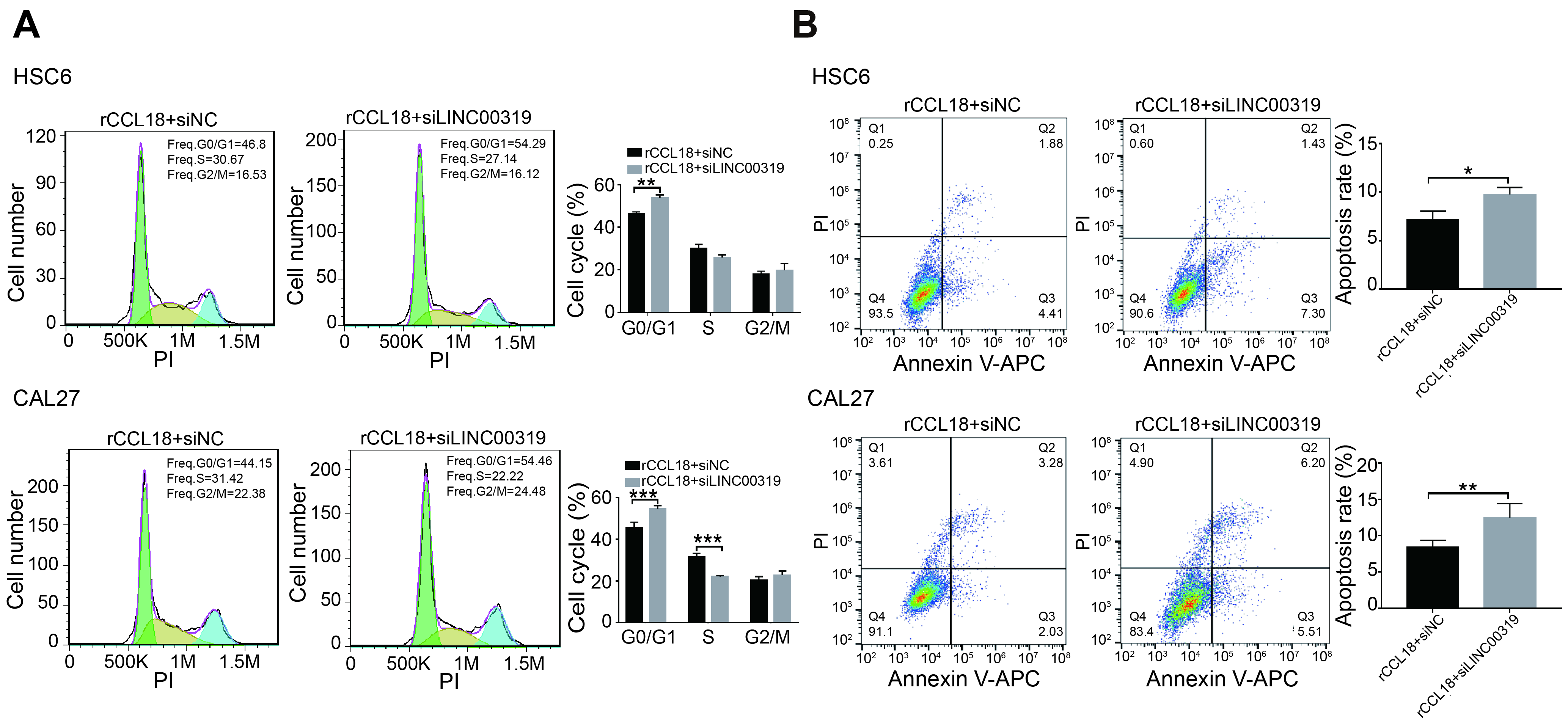

Supplement: Supplementary file 2 — Supplementary Figure1 [file 41419_2020_2978_MOESM2_ESM.tif]

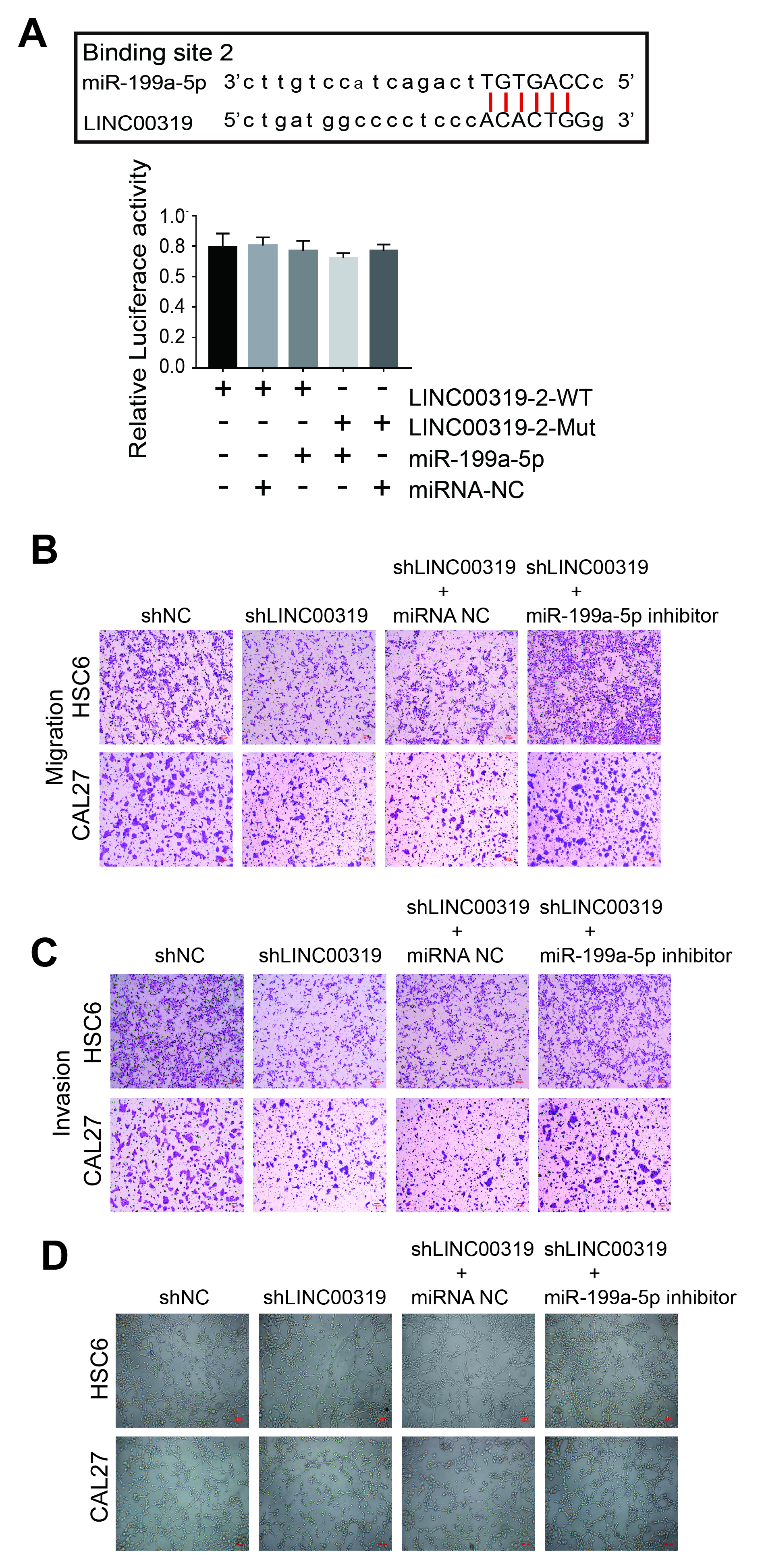

Supplement: Supplementary file 3 — Supplementary Figure2 [file 41419_2020_2978_MOESM3_ESM.tif]

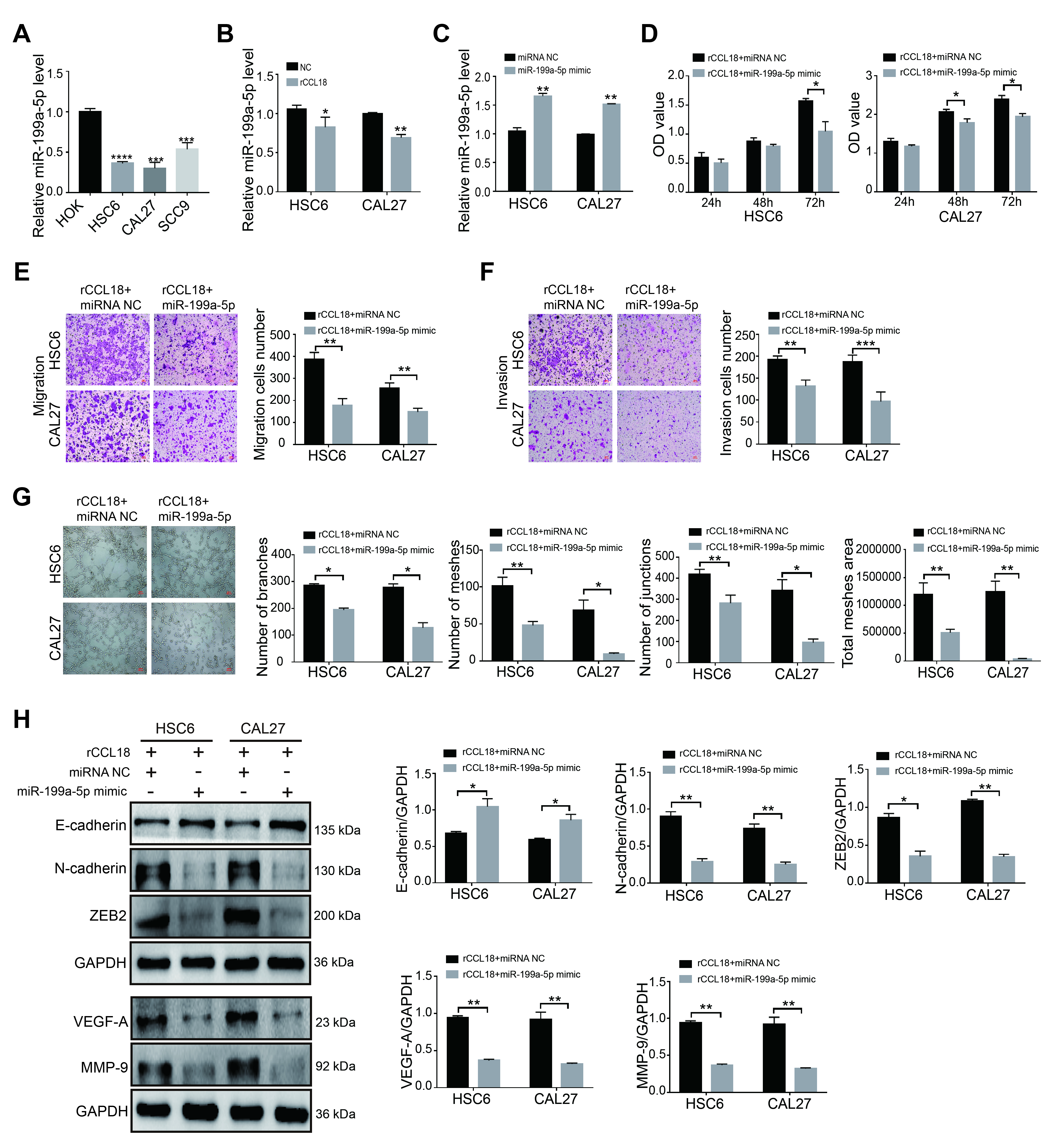

Supplement: Supplementary file 4 — Supplementary Figure3 [file 41419_2020_2978_MOESM4_ESM.tif]

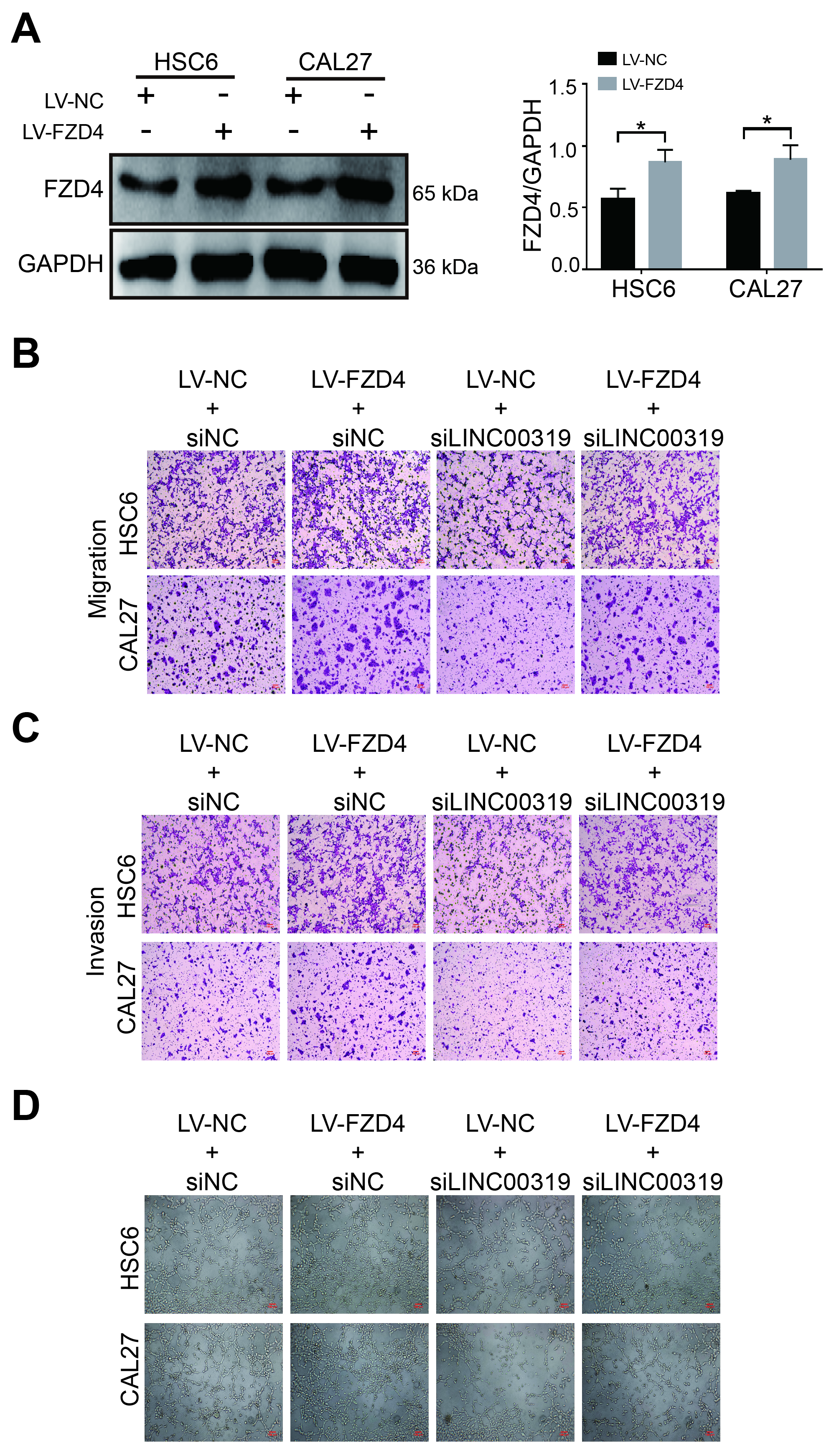

Supplement: Supplementary file 5 — Supplementary Figure4 [file 41419_2020_2978_MOESM5_ESM.tif]

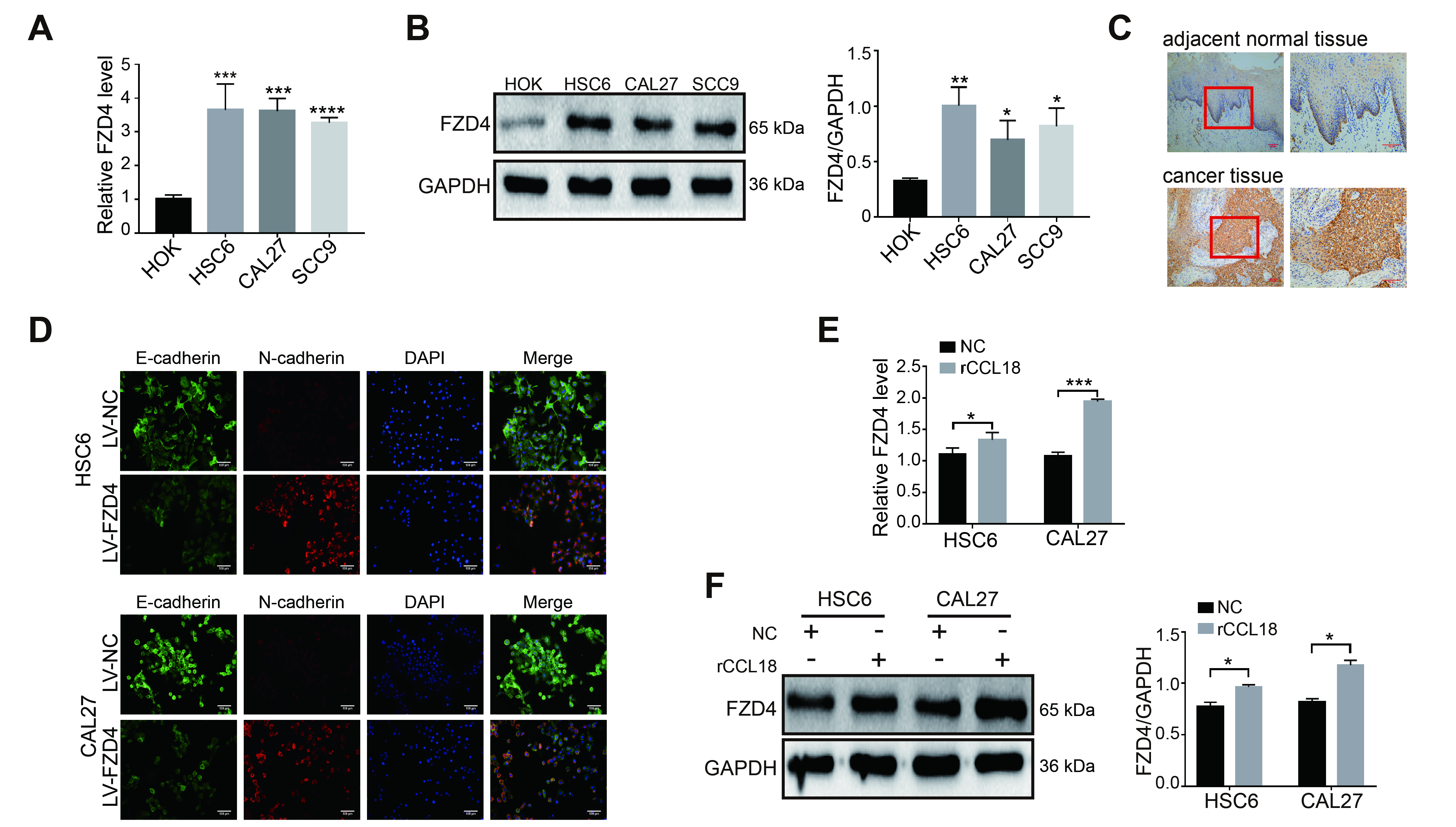

Supplement: Supplementary file 6 — Supplementary Figure5 [file 41419_2020_2978_MOESM6_ESM.tif]
