# Supplementary material for: CCL18-induced LINC00319 promotes proliferation and metastasis in oral squamous cell carcinoma via the miR-199a-5p/FZD4 axis
Source: Cell Death Dis. 2020 Sep 18;11(9):777. doi: 10.1038/s41419-020-02978-w (PMC7501282; doi:10.1038/s41419-020-02978-w)
Supplement: Supplementary file 7 — Supplementary Table 1 [file 41419_2020_2978_MOESM7_ESM.docx]

**Supplementary Table 1**

The sequence of siLINC00319, miR-199a-5p mimic and inhibitor

| Gene name | Sequence 5’-3’ |
| --- | --- |
| LINC00319-siRNA1 sense | GCTGTAATGTGCTGTGACT |
| LINC00319-siRNA1 antisense | AGTCACAGCACATTACAGC |
| LINC00319-siRNA2 sense | CCTTATGGAAGCCGGATAA |
| LINC00319-siRNA2 antisense | TTATCCGGCTTCCATAAGG |
| miR-199a-5p mimic sense | CCCAGUGUUCAGACUACCUGUUC |
| miR-199a-5p mimic antisense | ACAGGUAGUCUGAACACUGGGUU |
| miR-199a-5p mimic NC sense | UUCUCCGAACGUGUCACGUTT |
| miR-199a-5p mimic NC antisense | ACGUGACACGUUCGGAGAATT |
| miR-199a-5p inhibitor | GAACAGGUAGUCUGAACACUGGG |
| miR-199a-5p inhibitor NC | CAGUACUUUUGUGUAGUACAA |
