# Supplementary material for: CCL18-induced LINC00319 promotes proliferation and metastasis in oral squamous cell carcinoma via the miR-199a-5p/FZD4 axis
Source: Cell Death Dis. 2020 Sep 18;11(9):777. doi: 10.1038/s41419-020-02978-w (PMC7501282; doi:10.1038/s41419-020-02978-w)
Supplement: Supplementary file 8 — Supplementary Table 2 [file 41419_2020_2978_MOESM8_ESM.docx]

**Supplementary Table 2**

The antibodies used for western blot, immunofluorescence, and immunohistochemistry

| Antibody | Vendor and catalog number | Working Concentration Dilutions |
| --- | --- | --- |
| E-cadherin | Cell signaling (#3195) | WB: 1/1000, IF:1/200, IHC: 1/500 |
| N-cadherin | Santa Cruze (sc-393933) | WB: 1/1000, IF: 1/200, IHC:1/500 |
| ZEB2 | Merck (#ABT332) | WB: 1/1000, IHC: 1/50 |
| VEGF-A | Abcam (ab46154) | WB:1/1000, IHC: 1/200 |
| MMP-9 | Cell Signaling (#13667) | WB: 1/1000, IHC: 1/200 |
| FZD4 | R&D (#145901) | WB: 1/500, IHC: 1/200 |
| GAPDH | Abcam (ab8245) | WB: 1/2000 |
| Alexa Fluor 594 Goat anti-Mouse IgG | Telenbiotech (TL-W150) | IF: 1/200 |
| Alexa Flour 488 Goat anti-Rabbit IgG | Telenbiotech (TL-W801) | IF:1/200 |
| Goat anti-rabbit IgG-HRP | Asbio (AS006) | WB: 1/2000 |
| Horse anti-mouse IgG-HRP | Asbio (AS007) | WB:1/2000 |
