# Supplementary material for: CCL18-induced LINC00319 promotes proliferation and metastasis in oral squamous cell carcinoma via the miR-199a-5p/FZD4 axis
Source: Cell Death Dis. 2020 Sep 18;11(9):777. doi: 10.1038/s41419-020-02978-w (PMC7501282; doi:10.1038/s41419-020-02978-w)
Supplement: Supplementary file 9 — Supplementary Table 3 [file 41419_2020_2978_MOESM9_ESM.docx]

**Supplementary Table 3**

The primer of gene in the qRT-PCT

| Gene name | Sequence 5’-3’ |
| --- | --- |
| LINC00319-F | TGGAAGCCGGATAAGCACCT |
| LINC00319-R | GTTCATTGCCTGCACTTCGG |
| LINC00649-F | AATCAGCGAGACTCCGTGG |
| LINC00649-R | CTGGGCGTCTTCAGGTTGT |
| RP13-516M14.1-F | GATGCCATCAAGACAAATAGAAC |
| RP13-516M14.1-R | AGGGAAAGACGCCTTACACAA |
| RP11-454K7.1-F | AGGGGCTGCTGAGTGGTTC |
| RP11-454K7.1-R | GCTCCGATGGTGGGACTTC |
| miR-199a-5p-RT | CTCAACTGGTGTCGTGGAGTCGGCAATTCAGTTGAGGAACAGGT |
| miR-199a-5p-F | GCCGAGCCCAGTGTTCAGAC |
| miR-199a-5p-R | CTCAACTGGTGTCGTGGA |
| let-7a-5p-RT | CTCAACTGGTGTCGTGGAGTCGGCAATTCAGTTGAGAACTATAC |
| let-7a-5p-F | GCCGAGTGAGGTAGTAGGTT |
| let-7a-5p-R | CTCAACTGGTGTCGTGGA |
| FZD4-F | GAACCTCGGCTACAACGTGA |
| FZD4-R | GACTCTCTGGCCAGGCAAAT |
| FZD6-F | CATGTGGTTCCACCTTGTCG |
| FZD6-R | TTCAAGCTCCTCAGGCCATC |
| HOXA7-F | AGGAGTTCCACTTCAACCGC |
| HOXA7-R | CAGTCGGACCTTCGTCCTTA |
| ETS1-F | AGTGGTGAGGCAAGGACCTA |
| ETS1-R | CCCAAAAGGGGTAGCAAGGT |
| CTNNA2-F | AGCAGCTGTCAACTCACCTG |
| CTNNA2-R | AACCTCGTCGAACTCGTGTC |
| LAMC1-F | CGGAAAGTGCCAGTGAATGC |
| LAMC1-R | CCATGGCCAGTGGAACGATA |
| DUSP14-F | CATGGCCCCTCGGATGATTT |
| DUSP14-R | AATGCAGGTGATGCCACGA |
